# Supplementary material for: Anomalous thickness dependence of the vortex pearl length in few-layer NbSe2
Source: Nat Commun. 2025 Mar 31;16:2696. doi: 10.1038/s41467-025-57817-3 (PMC11958829; doi:10.1038/s41467-025-57817-3)
Supplement: Supplementary file 1 — Supplementary Information [file 41467_2025_57817_MOESM1_ESM.pdf]

Supplementary Information

Anomalous Thickness Dependence of the Vortex Pearl Length in Few-Layer $NbSe_2$

Nofar Fridman[1,2*], Tomer Daniel Feld[1,2], Avia Noah[1,2,3],Ayelet Zalic[1,2], Maya Markman[1,2],T.R Devidas[1,2], Yishay Zur[1,2], Einav Grynszpan[1,2], Alon Gutfreund[1,2], Itai Keren[1,2], Atzmon Vakahi[2], Sergei Remennik[2], Kenji Watanabe[4], Takashi Taniguchi[5] , Martin Emile Huber[6], Igor Aleiner[7], Hadar Steinberg[1,2], Oded Agam[1]*, Yonathan Anahory[1,2]*

[1] The Racah Institute of Physics, The Hebrew University, Jerusalem, 9190401, Israel
[2] Center for Nanoscience and Nanotechnology, Hebrew University of Jerusalem, Jerusalem, 9190401, Israel
[3] Faculty of Engineering, Ruppin Academic Center, Emek-Hefer, 40250 Monash, Israel
[4] Research Center for Electronic and Optical Materials, National Institute for Materials Science, 1-1 Namiki, Tsukuba 305-0044, Japan
[5] Research Center for Materials Nanoarchitectonics, National Institute for Materials Science, 1-1 Namiki, Tsukuba, Japan
[6] Departments of Physics and Electrical Engineering, University of Colorado Denver, Denver, CO 80217, USA
[7] Google Quantum AI, Santa Barbara, CA, USA
* Corresponding authors
nofarfri.friedman@mail.huji.ac.il ,yonathan.anahory@mail.huji.ac.il, agam.oded@gmail.com

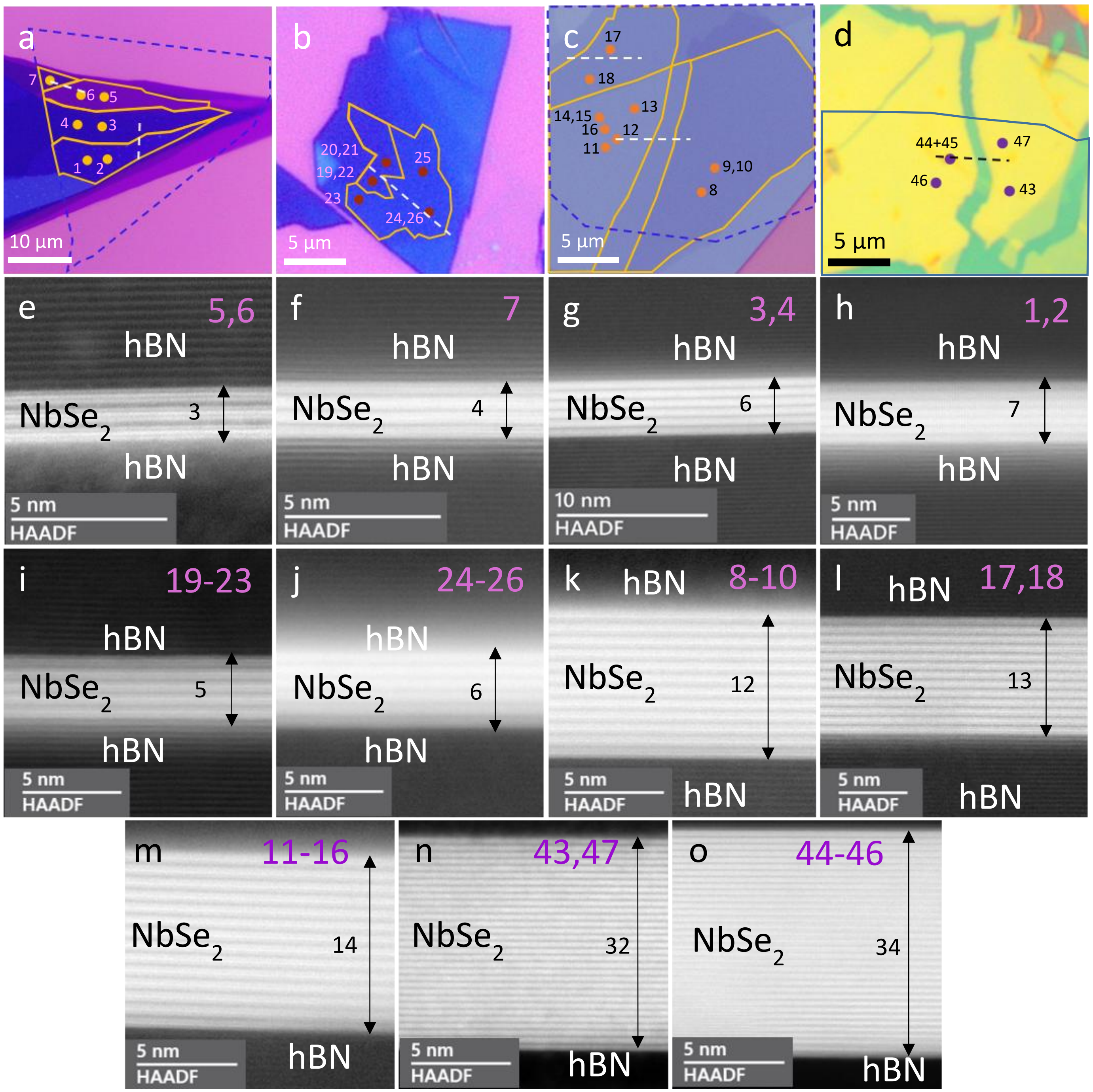

Supplementary Figure 1. Cross-sectional TEM images

(**a**-**d**) Optical images of the $NbSe_2$ thin flakes measured in this work. Dots on the images indicate the positions of the measured vortices, with numerical labels used to identify the vortex in further figures. Yellow outlines delineate the edges of the $NbSe_2$ terraces. White and black dashed lines in **a**-**c** and **d**, respectively, indicate the sections chosen for TEM cross-sectional analysis. In panels **a** and **c**, a dashed blue line demarcates the region of double encapsulation by hBN. (**b**) The entire field of view is encapsulated with top and bottom hBN. (**d**) Image of an encapsulated $NbSe_2$ in which the yellow region denotes the doubly encapsulated region, and the blue line shows the edge of the $NbSe_2$ flake. (**e**-**o**) Cross-sectional TEM images of the $NbSe_2$ flakes shown in **a**-**d** along the dashed lines. The numbers in purple correspond to the vortex labels in panels **a**-**d**. (**e**-**h**) TEM images taken from the sample shown in **a**, containing $N = 3, 4, 6$ and 7 layers, respectively. (**i**-**j**) TEM images taken from the sample shown in **b**, containing $N = 5$ and 6 layers, respectively. (**k**-**m**) TEM images taken from the sample shown in **c**, containing $N = 12, 13$ and 14 layers, respectively. (**n**-**o**) TEM images taken from the sample shown in **d**, containing $N = 32$ and 34 layers, respectively.

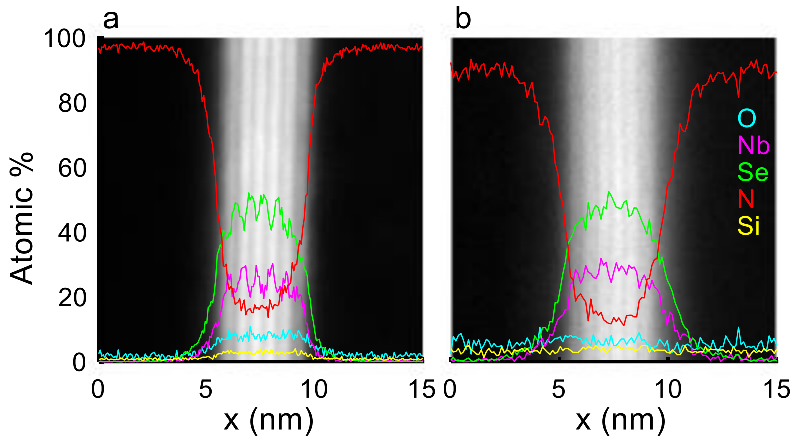

Supplementary Figure 2 Energy-dispersive X-ray spectroscopy chemical composition profile.

(**a**-**b**) Typical profile of the relative atomic percentage of the following elements Nb,Se,N,O and Si, corresponding to the cross sections shown in Supplementary Figure 1.a-b, respectively. Oxygen and nitrogen contamination is visible throughout the layers. Oxygen contamination is attributed to the air exposure upon lamella preparation while nitrogen is attributed to the layer intermixing caused by the Ga FIB during the lamella preparation. No surface contamination was detected.

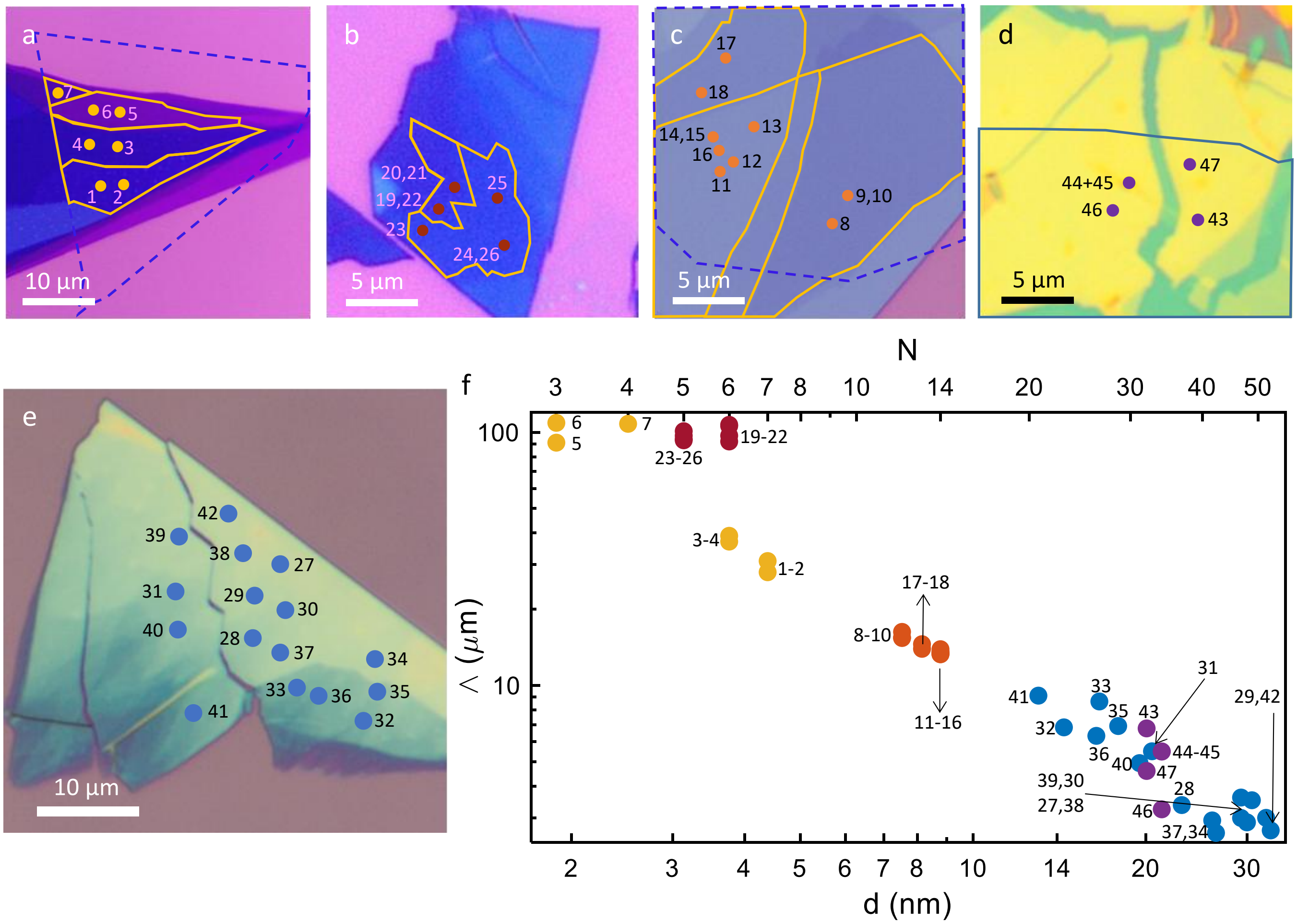

Supplementary Figure 3. Samples and Vortex Locations

(**a**-**e**) Optical images of all the flakes measured in this work. Dots mark the locations where vortices were imaged and numbers near them is the vortex number shown in **f**. (**a**,**c**) The edges of the terraces are delineated by yellow lines, and the dashed blue lines show the sample's area covered with top and bottom hBN.(**b**) The entire field of view is encapsulated with top and bottom hBN. (**d**) Image of an encapsulated $NbSe_2$. The yellow region is the doubly encapsulated region, and the blue line shows the edge of the $NbSe_2$ flake. (**e**) $NbSe_2$ flake that was not encapsulated. (**f**) The measured values of the Pearl length Λ as a function of the sample thickness d for all vortices shown in this work. The number near each data point is the vortex number shown in images **a**-**e**. Point color relates to the optical image from which the data point was acquired; yellow **a**, red **b**, orange **c**, purple **d**, and blue **e**.

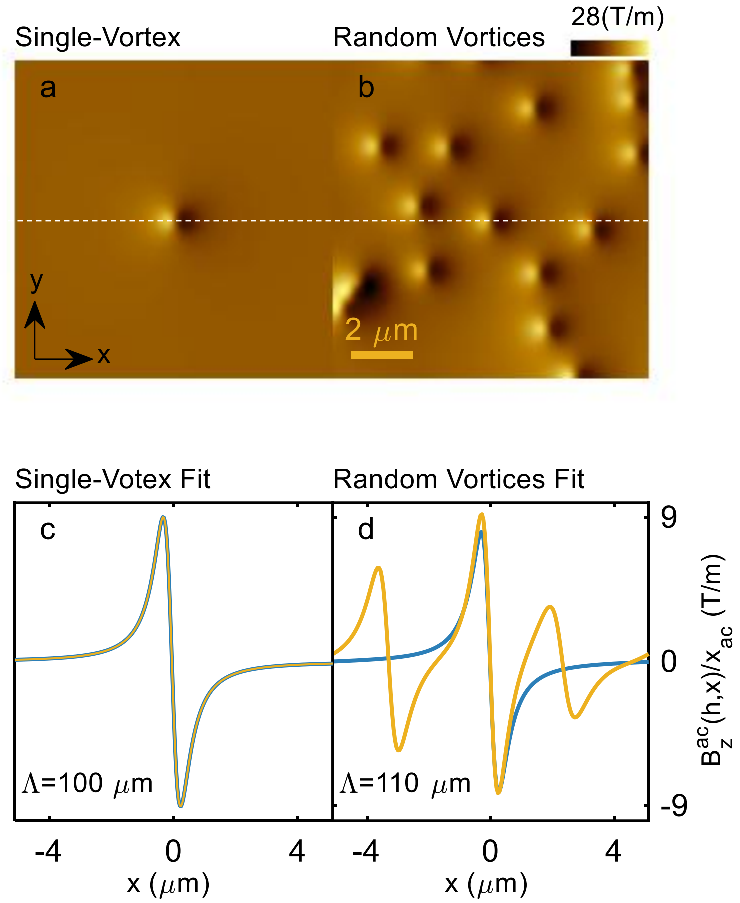

Supplementary Figure 4. The influence of nearby vortices on the Pearl length Λ

(**a**,**b**) Calculated theoretical spatial derivative along the x axis of the out-of-plane component of magnetic field $\frac{dB_z(h,\boldsymbol{r})}{dx}$ of vortices with Pearl length of $\Lambda = 100$ μm, where $h = 360$ nm is the distance from the tip-to-surface distance and $\boldsymbol{r} = (x, y)$ is the in-plane coordinate. The white dashed line marks the location of the cross-sections shown in panels **c**,**d**. (**a**) The analytical gradient of the magnetic field for a single vortex. (**b**) Multi-vortex simulation with average vortex spacing of 2 μm. (**c**,**d**) Cross-sections of the calculated magnetic field derivative along the white dashed line shown in panels **a**,**b** and their respective fits. The single-vortex fit gives $\Lambda = 100$ μm, while the fit for the multi-vortex situation yields $\Lambda = 110$ μm. Thus, the change in the Pearl Length due to nearby vortices is around 10%.

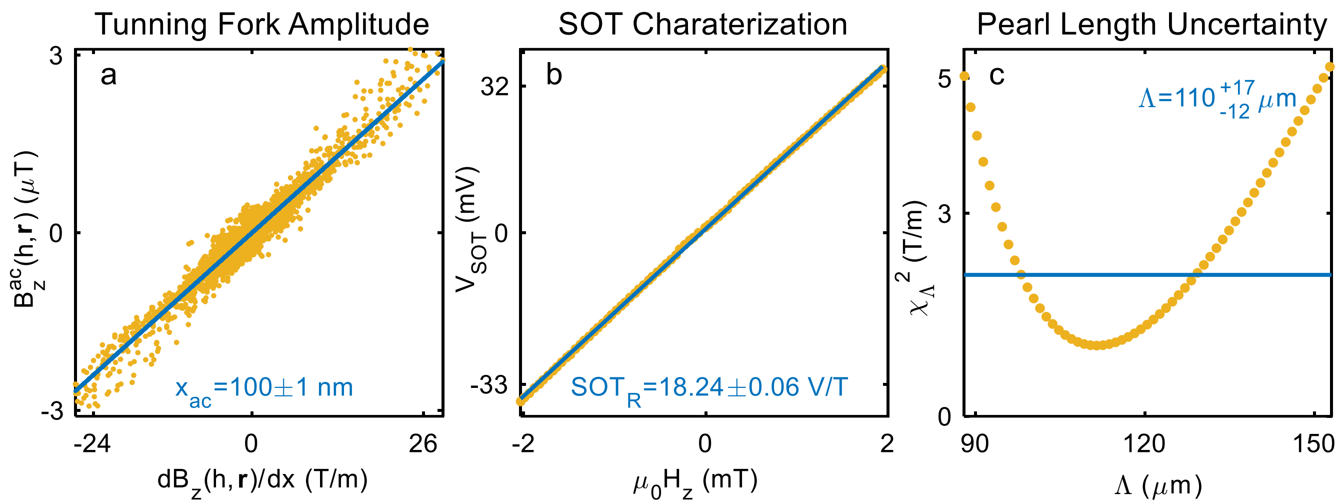

Supplementary Figure 5. Measurement error sources

(**a**) Tuning fork amplitude determination: A scatter plot of the data for the out-of-plane component of magnetic field $B_z^{ac}(h,\boldsymbol{r})$ and $\frac{dB_z(h,r)}{dx}$ (yellow dots) together with the best fit for the oscillation amplitude x_{ac} (blue line) as discussed in Supplementary Note 2, see Eq.(14). Here, h is tip-to-sample distance and $\boldsymbol{r} = (x, y)$ the inplane coordinate (**b**) Determination of SOT response coefficient - SOT_R. The measured voltage as a function of the magnetic field (yellow dots) and corresponding linear fit (blue line). The slope is SOT_R. (**c**) An example for the error analysis performed for vortex No. 6 shown in Supplementary Figure 3a,f, which corresponds to $N = 3$ layers. This panel shows the residual difference between modeled and measured image χ_Λ^2 as a function of the Pearl Length values (yellow dots) as discussed in Supplementary Note 3 and Eq. (13). $\chi_\Lambda^2 = 2 \cdot \chi_{\Lambda\min}^2$ (blue line), which determines one standard deviation σ_Λ.

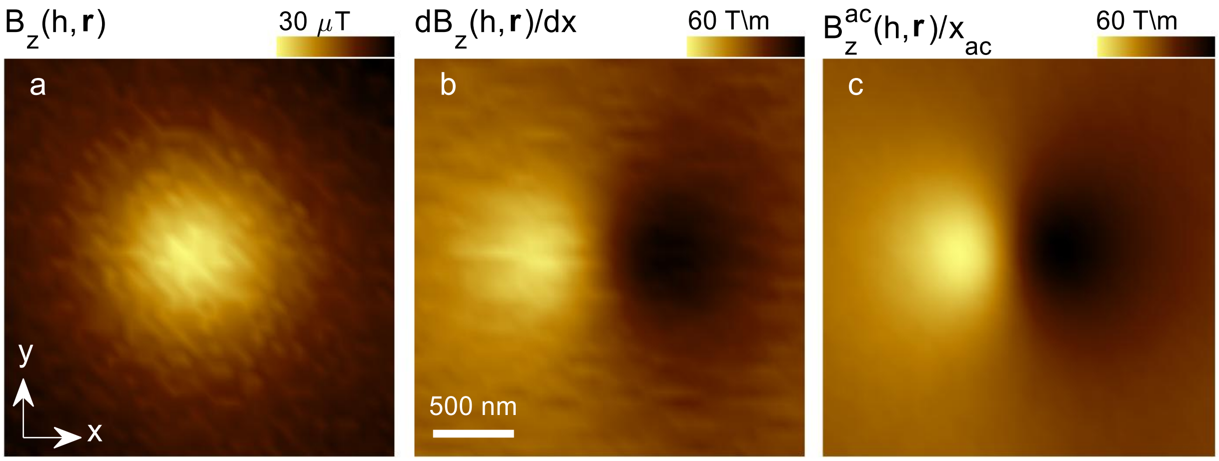

Supplementary Figure 6. Determination of the tuning fork amplitude

(**a**) SQUID-on-tip (SOT) image of the out-of-plane component of the magnetic field $B_z(h, \boldsymbol{r})$ of an isolated vortex located in the $N = 7$ layer region shown in Supplementary Figure 3a,f and referred as vortex No. 2. Here h is the tip-to-sample distance and $\boldsymbol{r} = (x, y)$ is the in-plane coordinate. This image was obtained at $\mu_0 H_z = 60$ μT with $h = 360$ nm. The signal from this vortex ($\Lambda = 30$ μm) is sufficiently large to measure in dc mode allowing us to determine the oscillation amplitude of the SQUID loop x_{ac} .(**b**) Numerical derivative of **a** along the x axis $\frac{dB_z(h,\boldsymbol{r})}{dx}$. (**c**) The in-phase component of the magnetic field oscillating at the tuning fork frequency $B_z^{ac}(h, \boldsymbol{r})$ and divided by the oscillation amplitude x_{ac}.This amplitude is the factor between the signals presented in panels **b** and **c**, and is determined through the scatter plot presented in Supplementary Figure 5a.

Supplementary Note 1: Magnetostatics of Finite Superconducting Films

In this section, our aim is to justify the approximations employed for the quantitative analysis of the magnetic field distribution. The main assumption hinges on the sample's size, L, being significantly smaller than the Pearl length, Λ, while also being much larger than the distance, h, between the squid tip and the sample surface.

Excluding the region of the superconducting film, which is assumed to be situated on the plane $z=0$ and its thickness is disregarded, a static magnetic $\boldsymbol{B}$ satisfies the Maxwell equations in vacuum:

$$\nabla\cdot\boldsymbol{B}=0 \quad \text{and} \quad \nabla\times\boldsymbol{B}=0. \tag{1}$$

However, the finite 2-dimensional current density J_α within the film, where $\alpha=x,y$ represents the coordinates on the film plane, causes a discontinuity in the planar components of the magnetic field, $\boldsymbol{B}$, across this plane:

$$\begin{aligned}\Delta B_x &= \mu_0 J_y,\\ \Delta B_y &= -\mu_0 J_x,\end{aligned} \tag{2}$$

where $\Delta\boldsymbol{B}=\boldsymbol{B}|_{z=0^+}-\boldsymbol{B}|_{z=0^-}$. It is convenient to define the dual counterpart of the current density vector, acquired through a 90-degree rotation, $\boldsymbol{J}^D=\hat{\boldsymbol{z}}\times\boldsymbol{J}$. This dual current density can be represented as $\boldsymbol{J}^D=\rho_s\boldsymbol{v}^D$, where ρ_s is the two-dimensional superfluid density and $\boldsymbol{v}^D$ is the dual superfluid velocity. Conservation of current implies that

$$\nabla\cdot\boldsymbol{J}=\nabla\times\boldsymbol{J}^D=0. \tag{3}$$

The dual superfluid velocity describes all quantum aspects of the superfluid current within the system:

$$\partial_\alpha v_\alpha^D = 2\pi\left[\sum_j \delta^{(2)}\left(\boldsymbol{r}-\boldsymbol{r}_j\right)-\frac{B_z}{\phi_0}\right]. \tag{4}$$

Here $\boldsymbol{r}_j$ are the position coordinates of the vortices in the film (all assumed to have the same vorticity), and ϕ_0 is the superconducting flux quantum. Equation (4) can be derived by variation of the Ginzburg-Landau energy functional after applying the Madelung transformation, $\psi=\sqrt{\rho_s}\exp(i\theta)$, and identifying the velocity as the gradient of the phase, $\boldsymbol{v}=\nabla\theta$. This equation expresses the fact that the circulation of the current arises solely from the presence of vortices or from the Aharonov-Bohm effect. If the film is of infinite size in the planar direction and the variations in ρ_s are neglected, a straightforward calculation of the Fourier transform of the component of the magnetic field that is perpendicular to the film,

$$\hat{B}_z(z,\boldsymbol{q})=\int d^2r\exp(-i\boldsymbol{q}\cdot\boldsymbol{r})B_z(z,\boldsymbol{r}) \tag{5a}$$

yields[2]

$$\hat{B}_z(z,\boldsymbol{q})=\hat{B}(\boldsymbol{q})\exp(-q|z|), \text{ with } \hat{B}(\boldsymbol{q})=\frac{\phi_0}{\Lambda q+1}n_V(\boldsymbol{q}), \tag{5b}$$

where $n_V(\boldsymbol{q})=\sum_j\int d^2r\exp\left(-i\boldsymbol{q}\cdot\boldsymbol{r}_j\right)$ is the Fourier transform of the vortex density, $q=|\boldsymbol{q}|$, and the Pearl length Λ is given by

$$\frac{1}{\Lambda}=\frac{\pi\rho_s\mu_0}{\phi_0}. \tag{6}$$

The finite size of the system presents a challenge when calculating the effects of the boundary currents, but at the same time, it allows for simplifying the problem. Specifically, when the magnetic field is sufficiently weak such that $\Lambda\gg L$, Eq. (4) can be approximated by

$$\partial_\alpha v_\alpha^D = 2\pi\sum_j \delta^{(2)}\left(\boldsymbol{r}-\boldsymbol{r}_j\right), \tag{7}$$

and neglecting variations of ρ_s within the sample implies that Eq. (3) reduces to

$$\nabla\times\boldsymbol{v}^D=0. \tag{8}$$

Furthermore, the condition for no direct current flow through the system's boundary translates into the condition that the dual velocity is perpendicular to the boundary - hereinafter denoted by Γ. Therefore, the solution of Eqs. (7) and (8) can be found from the solution for the two-dimensional Poisson's equation for the velocity potential, Φ :

$$\nabla^2\Phi = 2\pi\sum_j \delta^{(2)}\left(\boldsymbol{r}-\boldsymbol{r}_j\right) \quad \text{where} \quad v_\alpha^D = \partial_\alpha\Phi , \tag{9}$$

and with Dirichlet boundary conditions, $\Phi|_{r\in\Gamma} = 0$. The solution of this equation is given by:

$$\Phi(\boldsymbol{r}) = \sum_j \ln\left|\boldsymbol{r}-\boldsymbol{r}_j\right| - \oint_\Gamma dl\hat{\boldsymbol{n}}'\cdot\boldsymbol{v}^D(\boldsymbol{r}')\ln\left|\boldsymbol{r}-\boldsymbol{r}'\right| , \tag{10}$$

where dl is an infinitesimal length element along the boundary, while $\hat{\boldsymbol{n}}'$ is a unit vector normal to the boundary at point $\boldsymbol{r}'$ that resides on the boundary, and pointing outwards. The dual current on the boundary, $\boldsymbol{v}^D(\boldsymbol{r}')$, should be found from the solution of integral equation derived from Eqs. (8-10) with Dirichlet boundary conditions $\Phi|_{r\in\Gamma} = 0$. The solution of this equation is complicated for the sample shapes used in the experiments. Nevertheless, for the interpretation of the experimental data, $\boldsymbol{J}^D(\boldsymbol{r})$ is not required, as we explain below.

Solution of Eqs. (1-3) and (7-8) is explicitly given by a sum of two component:

$$B_z(h,\boldsymbol{r}) = B_z^{(1)} + B_z^{(2)} , \tag{11a}$$

where

$$B_z^{(1)} = \frac{\phi_0}{2\pi\Lambda}\sum_j \frac{1}{\sqrt{h^2+\left(\boldsymbol{r}-\boldsymbol{r}_j\right)^2}} , \tag{11b}$$

is the contribution from vortices, while

$$B_z^{(2)} = -\frac{\phi_0}{2\pi\Lambda}\sum_j \oint_\Gamma \frac{dl\hat{\boldsymbol{n}}'\cdot\boldsymbol{v}^D(\boldsymbol{r}')}{\sqrt{h^2+\left(\boldsymbol{r}-\boldsymbol{r}'\right)^2}} \tag{11c}$$

is the magnetic field generated by boundary currents. A comparison of the vortex contribution (11b) with the boundary term (11c) suggests a procedure by which the latter is eliminated. It is achieved by considering the gradients of $B_z(\boldsymbol{r})$ rather than the field itself. Indeed, estimation from Eqs. (11) yield

$$\frac{\max\left\|\partial_x B^{(2)}\right\|}{\max\left\|\partial_x B^{(1)}\right\|} \simeq \frac{h^2}{L^2} , \tag{12}$$

where L is the linear size of the system that satisfies the condition, $h \ll L \ll \Lambda$. Here it is assumed that the vortex distance from the boundary (or other vortices) is of order L. This result means that for the purpose of extracting the Pearl length from a χ^2 fit (described in the next section) one can ignore the boundary contribution and use only the contribution associated with a single vortex.

Supplementary Note 2: Quantitative Analysis of the Pearl Vortices

We employ the least squares method[1] to determine the Pearl length Λ from our SOT images. We numerically minimize the parameter χ^2 which is given by:

$$\chi^2 = \sum_{i=1}^{N_{\text{pixel}}} \left(\frac{dB_z\left(h, \boldsymbol{r}_i\right)}{dx} - \frac{dB_z^{th}\left(h, \boldsymbol{r}_i\right)}{dx} \right)^2 , \tag{13}$$

where $dB_z\left(h,\boldsymbol{r}\right)/dx$ is the spatial derivative of the out-of-plane component of the magnetic field emanating from the vortex, $dB_z^{th}\left(h,\boldsymbol{r}\right)/dx$ is the theoretical spatial derivative of the magnetic field calculated using the Pearl model, and the sum is over all the pixels of the image in which $\boldsymbol{r}_i$ denotes the position corresponding to the ith pixel.

The magnetic field derivative is directly measured by sensing the oscillating component of the magnetic field, $B_z^{ac}\left(h,\boldsymbol{r}\right)$, caused by an in-plane oscillation of the SQUID loop. We divide this signal by the oscillation amplitude x_{ac} to obtain the derivative. Using this measurement technique has many benefits, as discussed in the main text. The oscillation amplitude, x_{ac}, is determined using the following relation:

$$\frac{dB_z\left(h,\boldsymbol{r}\right)}{dx} \simeq \frac{B_z^{ac}\left(h,\boldsymbol{r}\right)}{x_{ac}} , \tag{14}$$

for an image with a strong gradient as illustrated in supplementary figure 6. This image can be, for example, a vortex with a $\Lambda \lesssim 30$ μm. We find the proportional constant that satisfies equation (14) by calculating numerically the gradient of the dc image $dB_z\left(h,\boldsymbol{r}\right)/dx$ (supplementary figure 6b) and comparing it to the measured $B_z^{ac}\left(h,\boldsymbol{r}\right)$ using a lock-in amplifier (Supplementary Figure 6c). For a given set of measurements, the oscillation amplitude is constant within 2%. The difference between measurement and the theoretical formula in Eq. (13) is performed for every pixel for the entire image containing N_{pixel} pixels.

The starting point for calculating $dB_z^{th}\left(h,\boldsymbol{r}\right)/dx$ is the formula for the magnetic field of a single Pearl vortex, which in Fourier space is given by Eq. (5b) with $n_V\left(\boldsymbol{q}\right)=1$. This expression is transformed into real space and differentiate with respect to x. The resulting expression is calculated for the same number and size of pixels as our SOT image. This analysis yields the Pearl length values for all the vortices, plotted as a function of thicknesses, as shown in Figure 4.

Supplementary Note 3: Quantitative Analysis of Pearl Length Uncertainties

There are two relevant measured quantities in this work: the sample thickness N and the Pearl length Λ. In the following, we estimate their respective uncertainties.

3.1 Uncertainty on the Pearl length Λ

The following uncertainty estimation takes into consideration the uncertainty inherent to each measured parameter required to compare the measured signal (in volts) with the model (section 3.1.1 below). Another contribution to the uncertainty is related to the signal-to-noise ratio and other imaging artefacts (section 3.1.2 below).

3.1.1 Input parameter uncertainty

The delta method[3] serves to evaluate the uncertainty on the Pearl Length given the uncertainty on other measured parameters. The method relies on prior measurements of each parameter s_i, and its uncertainty σ_{s_i}. These parameters are the SQUID Tesla-to-Volt transfer function SOT_R, the SQUID loop in-plane oscillation amplitude x_{ac} and the distance between the SQUID loop and the surface of the sample h. To calculate the resulting uncertainty on Λ, each parameter is individually perturbed by one standard deviation $\pm\sigma_{s_i}$ as follows:

$$\Delta\widehat{\Lambda}_1 = \widehat{\Lambda}(s_1 \pm \sigma_1, s_2, s_3) - \widehat{\Lambda}(s_1, s_2, s_3). \tag{15}$$

Here $\Delta\widehat{\Lambda}_1$ represents the uncertainty arising from perturbing s_1. The total uncertainty on Λ is obtained by summing in quadrature the uncertainty resulting from each parameter:

$$\left(\Delta\widehat{\Lambda}_{tot}\right)^2 = \sum_i \left(\Delta\widehat{\Lambda}_i\right)^2 . \tag{16}$$

We now discuss the uncertainty evaluation of each relevant parameter.

The transfer function SOT_R is determined by calibrating the SQUID voltage response to a known change in the magnetic field (Supplementary Figure 5b). The uncertainty on this parameter is caused by statistical fluctuations in the measurement, as shown in Supplementary Figure 5b. For example, the calibration obtained while measuring vortex 6 has an estimated uncertainty of 2%, which translates into an uncertainty of 2 μm (2%) in the measurement of $\Lambda = 111$ µm.

The method used to determine x_{ac}, and its associated uncertainty is described in Supplementary Note S2 and shown in Supplementary Figure 5a. The uncertainty on this parameter is caused by statistical fluctuations in the measurement, as shown in Supplementary Figure 5a. For example, the uncertainty on x_{ac} for vortex 6 is estimated to be 1%, which corresponds to 1 μm (1%) uncertainty in the measurement of Λ.

The distance between the tip and sample h is determined by employing the tuning fork to sense the sample surface, akin to an AFM operation. We approximate its uncertainty to be ± 15 nm, factoring the uncertainty on the thickness of the hBN layer covering the sample and other surface impurities (10 nm). We also consider the statistical variance of the surface sensing, which exhibited variations within ± 10 nm. This uncertainty for vortex 6 translates into $\Lambda = 111^{+8}_{-7}$ µm.

The total uncertainty is calculated for each vortex separately and can vary, but they are typically around ~9%, and the main contribution is the uncertainty on h.

3.1.2 Uncertainty caused by the image signal-to-noise ratio and other imaging artefacts

As explained in note 2, the least squares method minimizes the difference between the magnetic profile of the modeled and the measured vortex. Given the noise in our measurements and other imaging artifacts discussed below, the smallest residual is $\chi^2_{min} > 0$. We calculate the interval of confidence for Λ (within one standard deviation $\pm\sigma$) by considering the range of Λ for which $\chi^2 < 2 \times \chi^2_{min}$.[1] Supplementary Figure 5c shows a typical example of χ^2 as a function of Λ for a particular vortex (vortex number 6). From this graph, we estimate the uncertainty, which typically is +16/-11 % for vortices of low signal ($N < 10$).

Another uncertainty stems from imaging artefacts that are unaccounted in the Pearl model. We consider two main sources of unaccounted background. The presence of vortices and the presence of edges near the vortex of interest. These two sources have the same effect but with opposite sign given that the boundary conditions caused by the edge is modeled by a mirror image vortex. The influence of nearby vortices is shown in Supplementary Figure 4a-d where we simulate a sample with $\Lambda = 100$ µm in presence of other vortices at the same distance we would encounter in a real experiment. The result is an enlargement of Λ of typically 10%. Consequently, edges located at the same distance from the vortex of interest would have the opposite effect of shrinking Λ by 10%.

3.1.3 Uncertainty on the tip diameter

The uncertainty of the tip diameter influences the measured Λ because the image resulting from the Pearl model is convoluted with the tip size to account for finite tip-size smoothing. We note that the convolution method is only sensitive to a change in diameter greater than the pixel size (60 nm). The tip diameter was determined by measuring the field period of the SQUID interference pattern ΔH_z. Knowing that one period corresponds to a change in flux corresponding to the flux quantum Φ_0, we can state that $\pi r^2 \Delta H_z = \Phi_0$. The uncertainty of the field period is small (a few percent). In principle, other effects, such as flux focusing, could change the effective diameter. However, such an effect was never observed on a magnitude comparable with the pixel size (Ref. 16 and 17 of the main text). The negligible influence is most likely due to the thin film geometry found in the SOT. For those reasons, we consider the uncertainty on the tip diameter as negligible.

3.1.4 Summing all uncertainties

Here we present a table of the average parameter uncertainties and their resultant Pearl uncertainties:

	**Input Parameters**			**Fitting Uncertainty**	**Environment Artefacts**	
**Parameter Names**	SOT_R	x_{ac}	h	Λ	Boundary Conditions	Surrounding Vortices
**Parameter Uncertainty %**	±2	±2	±5			
**Resultant Pearl Positive Uncertainty %**	2	2	9	16	0	10
**Resultant Pearl Negative Uncertainty %**	2	2	8	11	10	0

Finally, the sum of all the uncertainties takes the following form:

$$\Delta_{\Lambda tot}^{Lower\ Boundary} = \sqrt{\Delta^2_{Input\ ParametersLB} + \Delta^2_{\Lambda\mathrm{LB}} + \Delta^2_{\mathrm{surrounding\ verticesLB}} + \Delta^2_{\mathrm{boundary\ conditionsLB}}} = \sqrt{8^2 + 12^2 + 0^2 + 11^2} = 18\ \mu m$$

$$\Delta_{\Lambda tot}^{Upper\ Boundary} = \sqrt{\Delta^2_{Input\ ParametersUB} + \Delta^2_{\Lambda\mathrm{UB}} + \Delta^2_{\mathrm{surrounding\ verticesUB}} + \Delta^2_{\mathrm{boundary\ conditionsUB}}} = \sqrt{9^2 + 17^2 + 11^2 + 0^2} = 22\ \mu m$$

$\Delta_{\Lambda tot}^{Lower\ Boundary}$ and $\Delta_{\Lambda tot}^{Upper\ Boundary}$ indicate the lower and upper limits. As, the presence of neighboring vortices, results in a positive deviation thus, they contribute to the upper limit. Conversely, the boundary conditions contribute as negative vortices and are thus relevant to the lower limit. The final result here is:

$$\Lambda_6 = 111^{+22}_{-18}\ \mu m$$

The same type of calculation is conducted for each vortex and gives the error bars shown in Fig. 4. Λ_6 depicts the full uncertainty calculation for a vortex residing in the 3 layer region. The average uncertainties of Pearl lengths are given in the table above.

3.2 Uncertainty of the sample thickness

Finally, we address the evaluation of thickness uncertainties. The flakes under consideration, with the exception of the one featured in Supplementary Figure 3e, exhibited thickness uniformity across the measurement steps, as ascertained via TEM (Supplementary Figure 1), resulting in no uncertainties.

For the specific flake showcased in Supplementary Figure 3e, thicknesses were determined using AFM images. This particular flake displayed less uniform steps and was not covered with hBN, leading to associated thickness uncertainties. To gauge these uncertainties, AFM scans were conducted over a region of approximately 1 μm in diameter surrounding the vortex location. The assigned thickness was established as the average thickness, and its uncertainty is assigned as the standard deviation $d_i + \Delta d_i$. For this sample, the typical uncertainty in thickness was 10%.

Supplementary References

1. James, G., Witten, D., Hastie, T., Tibshirani, R. & Taylor, J. *An Introduction to Statistical Learning*. Springer International Publishing, 72-78 (2023).
2. Pearl, J. Current distribution in superconducting films carrying quantized fluxoids. *Appl. Phys. Lett* **5**, 65 (1964).
3. Cox, C. Fieller's Theorem, the Likelihood and the Delta Method. *Biometrics* **46**, 709 (1990).
